# Prevalence of *Escherichia coli* O157:H7 in beef cattle at slaughter and beef carcasses at retail shops in Ethiopia

**DOI:** 10.1186/s12879-017-2372-2

**Published:** 2017-04-17

**Authors:** Rosa Abdissa, Woynshet Haile, Akafete Teklu Fite, Ashenafi Feyisa Beyi, Getahun E. Agga, Bedaso Mammo Edao, Fanos Tadesse, Mesula Geloye Korsa, Takele Beyene, Tariku Jibat Beyene, Lieven De Zutter, Eric Cox, Bruno Maria Goddeeris

**Affiliations:** 1 College of Veterinary Medicine and Agriculture, Addis Ababa University, Bishoftu, Oromia Ethiopia; 2 Department of Animal Sciences, University of Florida, Gainesville, Florida, USA; 3 U.S. Department of Agriculture, Agricultural Research Service, Food Animal Environmental Systems Research Unit, Bowling Green, KY USA; 4 Faculty of Veterinary Medicine, University of Ghent, Ghent, Belgium; 5 Division Animal and Human Health Engineering, Katholieke Universiteit Leuven, Leuven, Belgium; 6 The Asia-Pacific Centre for Animal Health, Faculty of Veterinary and Agricultural Sciences, the University of Melbourne, Parkville, VIC Australia; 7 Business Economics Group, Wageningen University, Wageningen, The Netherlands

**Keywords:** *E. coli* O157:H7, Beef cattle, Skins, Carcass, Antimicrobial resistance

## Abstract

**Background:**

There is paucity of information regarding the epidemiology of *Escherichia coli* O157: H7 in developing countries. In this study, we investigated the occurrence of *E. coli* O157: H7 associated with beef cattle at processing plants and at retail shops in Ethiopia.

**Methods:**

Various samples were collected from beef cattle at slaughter/processing plants, carcass at retail shops and humans at health centers. *E. coli* O157: H7 was isolated, identified and characterized for antimicrobial resistance, using standard microbiological methods.

**Results:**

At the processing plants *E. coli* O157: H7 was detected in 1.89% of fecal, 0.81% of intestinal mucosal swab, 0.54% of skin swab and 0.54% of carcass internal swab samples. At retail shops it was detected in 0.8% of carcass and 0.8% of cutting board swab samples, while all samples from utensils, hands from workers, and fecal and stool samples were negative. All isolates were resistant to Amoxicillin, moderately resistant to Cefoxitine and Nitrofurantoins but susceptible to other antimicrobials tested.

**Conclusions:**

*E. coli* O157: H7 occurs at low prevalence in beef cattle, and the current sanitary dressing procedures in the processing plants and storage conditions in the retail shops are effective against *E. coli* O157: H7.

## Background

Foodborne pathogens are the leading cause of illness and death in developing countries costing billions of dollars in medical care and social costs [1]. *E. coli* O157: H7, an enterohemorrhagic *E. coli* (EHEC), is one of the most common causes of foodborne infections in humans. It infects all age groups and the pathogen is noted for its severe consequences following infection, low infective dose and acid resistance [2]. Depending on the immune status and the general health of the infected individual, and the dose and virulence of the bacteria, infection with *E. coli* O157: H7 can result in mild diarrhea, severe bloody diarrhea, hemorrhagic colitis, or hemolytic uremic syndrome (HUS) leading to kidney failure [2, 3].

Cattle are the primary reservoirs of *E. coli* O157:H7 and ground beef and beef products are identified as major sources of foodborne transmission [2, 4]. Carcass contamination occurs through skin-to-carcass or fecal-to-carcass transfer of the pathogen during slaughter process at processing plants [5–7]; and this is the major risk factor for human infection. Furthermore, cross-contamination can occur during further processing of carcasses in the processing plants, during distribution and storage of beef at retail markets. Various pre-harvest interventions (vaccination, direct-fed microbial and bacteriophage treatment) to reduce pathogen shedding [8, 9] and post-harvest intervention technologies such as skin and carcass washing, and the use of antimicrobials [10] have been developed with varying success.

Despite greater burden caused by foodborne infections in developing countries than developed countries, there is acute scarcity of information on their occurrences [1]. In Ethiopia, only very few studies can be found regarding *E. coli* O157:H7 in animals, animal products or people [11–14]. Therefore producing more information on this particularly important foodborne pathogen is crucial to create awareness in the public and formulate preventive measures along food production, processing, and distribution continuum. The objective of this study was to investigate the occurrence of *E. coli* O157:H7 associated with beef cattle at processing plants, retail shops, and in people sampled at health centers in Ethiopia.

## Methods

### Sample collection

Various sample types were collected at two processing plants (anonymously designated as plant A and plant B), retail shops, and public health centers in Addis Ababa and Debre Berhan cities. Plant A is located in Addis Ababa and on average it processes about 700 heads of cattle per day. Plant B is located in Debre Berhan and it is relatively smaller compared to plant A, and on average it processes about 30 heads of cattle per day. At plant A samples were collected once per week and at plant B twice per week with 14 and 28 sampling occasions, respectively. At each visit to the plants, 10 to 15 animals were randomly selected and sampled. Five different types of samples from each animal (*n* = 370) and one pooled environmental swab sample (*n* = 62) were collected during each visit to the processing plants. Fecal, intestinal mucosal swab, skin swab, and carcass swab samples were collected according to previously described methods [7, 15]. For the collection of fecal and intestinal mucosal swab samples, the distal colon was ligated, transected proximal to the rectum and transported on ice to the laboratory. In the laboratory, each colon was aseptically opened and fecal samples were recovered. Intestinal lumen mucosa was swabbed by using cotton tipped swabs after extra fecal content was removed. The swabs were placed in test tubes containing 10 ml buffered peptone water (BPW; Oxoid Ltd., Hampshire, England).

Skin swabs were obtained immediately after exsanguination by using cotton tipped swabs presoaked in 10 ml of BPW by swabbing ≈10 × 10 cm area of the neck covering the bleeding line. Two carcass swab samples were obtained per animal to examine skin-to-carcass (external part of the carcass) and fecal-to-carcass (internal surface of carcass facing the gastrointestinal tract) transfer of *E. coli* O157:H7 during dressing and evisceration, respectively. The external part of the carcass was swabbed from the neck, brisket, flank and rump using cotton tipped swabs presoaked in 10 ml of BPW. The internal part of the carcass was swabbed from thoracic and pelvic parts as described above. In addition, pooled swab samples (referred to as environmental swabs) from knives, workers’ hands and aprons were collected on each abattoir visit. At retail markets, swab samples were collected from the carcass (*n* = 125), knife (*n* = 125), cutting board (*n* = 125), and workers’ hands (*n* = 125) and were placed in separate screw-capped tubes containing 10 ml BPW. Stool samples that were submitted to laboratories of the health centers with physicians’ orders to test for enteric pathogens were obtained at two health centers in Addis Ababa and at a referral hospital in Debre Berhan. Stool samples were collected from patients who were presented with diarrhea. All samples were kept in icebox and transported to the laboratory.

### Sample processing for prevalence

After the colon was aseptically opened 25 g of feces was transferred into a stomacher bag to which 225 ml of modified tryptone soya broth supplemented with 20 mg/l novobiocin (mTSB + n) was added for pre-selective enrichment. The resulting mixture was homogenized using a laboratory blender at low speed for 30 s. To all swab samples 90 ml of mTSB + n was added and the mixture was vortexed for 30 s. All the pre-enrichments were incubated at 41.5 °C for 24 h. Following the incubation period, 1 mL from each enrichment was subjected to anti-O157 immunomagnetic separation (IMS). Enrichments (1 mL) received 20 μL of anti-O157 beads (Dynal Biotech ASA, Oslo, Norway). The beads were extracted from the enrichment samples and washed three times in phosphate-buffered saline-Tween 20 (Sigma, St. Louis, MO). Fifty microliters of the final bead-bacteria complexes were spread-plated onto Sorbitol MacConkey agar (Oxoid Ltd., Hampshire, UK) containing 0.05 mg/l cefixime and 2.5 mg /l potassium tellurite (CT-SMAC; Dynal Biotech ASA). All plates were incubated at 37 °C for 24 h. After the plates were incubated, suspect colonies were picked and tested by an *E. coli* O157: H7 latex agglutination test (Oxoid Ltd., Hampshire, UK) following the manufacturer’s instruction.

### Antimicrobial susceptibility testing

All *E. coli* O157:H7 isolates were tested for susceptibility to a panel of 10 antimicrobial agents (Amoxicillin 25 μg, Kanamycin 30 μg, Trimethoprim-Sulfamethoxazole 25 μg, Chloramphenicol 30 μg, Ciprofloxacin 5 μg, Streptomycin 10 μg, Nalidixic acid 30 μg, Cefoxitin 30 μg, Tetracycline 30 μg and Nitrofurantoin 50 μg) by agar disc diffusion method according to Clinical and Laboratory Standards Institute [16] procedure. Briefly, isolated colonies were inoculated into tryptone soya broth (Oxoid Ltd., Hampshire, UK) and incubated for 6 h. The turbidity of the broth was adjusted to 0.5 McFarland standards (≈3 × 10^8^ CFU/ml) using sterile saline solution and inoculated on Muller-Hinton agar (Becton, Dickinson and Company, MD, USA) using sterile cotton swabs. Antimicrobial containing discs (Oxoid Ltd., Hampshire, UK) were applied to the surface of the agar plates, and the plates were incubated at 37 °C for 24 h. After incubation, the antimicrobial inhibition zone diameters were measured and results were qualitatively interpreted as susceptible, intermediate or resistant based on CLSI interpretative criteria. The antimicrobial agents included on the disc diffusion susceptibility panels and breakpoints used for the interpretation of results are listed in Table 1. *E. coli* ATCC 25922 was used as a quality control strain.

**Table 1:** Interpretative criteria for *E. coli* O157:H7 using disk diffusion susceptibility testing reported as inhibition zone diameters (mm)

Antimicrobial	Disk (μg)	Susceptible	Intermediate	Resistant	Antimicrobial class	WHO classification
Amoxicillin	25	≥17	14–16	≤13	β-lactams	Critically important
Cefoxitin	5	≥18	15–17	≤14	Cephalosporins	Critically important
Chloramphenicol	30	≥18	13–17	≤12	Phenicols	Highly important
Ciprofloxacin	5	≥31	21–30	≤20	Quinolones	Critically important
Kanamycin	30	≥18	14–18	≤13	Aminoglycosides	Critically important
Nalidixic acid	30	≥19	14–18	≤13	Quinolones	Critically important
Nitrofurantoin	300	≥17	15–16	≤14	Nitrofurans	Important
Streptomycin	10	≥15	12–14	≤11	Aminoglycosides	Critically important
Trimethoprim-sulfamethoxazole	1.25/23.75	≥16	11–15	≤10	Folate pathway inhibitors	Highly important
Tetracycline	30	≥15	12–14	≤11	Tetracyclines	Highly important

### Statistical analysis

Stata version 13 [17] was used for the statistical analysis. Prevalence was expressed as the percent positive samples from total samples tested. Differences in the prevalence of *E. coli* O157: H7 between the two processing plants (A and B), sample sources (processing plants, retail shops and health centers) and sample types (feces, skin, carcass or stool) were assessed by Fisher’s exact test. The significance level was set at α = 0.05.

## Results

### Prevalence of *E. coli* O157:H7

No statistically significant (*P* > 0.05) differences were observed between the two processing plants, sample sources or sample types. Therefore, results from the two processing plants were combined and are shown in Table 2. *E. coli* O157:H7 was detected in 2% of the fecal samples, 0.5% of the skin swabs, 0.8% of the intestinal mucosal swabs and 0.5% of the internal carcass swabs at the processing plants. At the retail shops, it was detected in 0.8% of carcass swabs and 0.8% of cutting board swabs examined. All samples collected from knives and workers both at the processing plants and retail shops, and stool samples obtained from clinically suspected people at the health centers were negative for *E. coli* O157:H7.

**Table 2:** Prevalence of *E. coli* O157:H7 from various samples collected at processing plants, retail markets and health centers in Ethiopia

Sample source	Sample type	No. of sample Examined	No. of positive	% Positive (95% CI^a^)
Processing plants	Fecal sample	370	7	1.89 (0.92, 3.85)
	Skin swab	370	2	0.54 (0.15, 1.95)
	Intestinal mucosal swab	370	3	0.81 (0.28, 2.26)
	Carcass internal swab	370	2	0.54 (0.15, 1.95)
	Carcass external swab	370	0	0
	Environmental swabs	62	0	0
Retail shops	Carcass	125	1	0.8 (0.14, 4.39)
	Hands	125	0	0
	Cutting board	125	1	0.8 (0.14, 4.39)
	Knife	125	0	0
Health centers	Stool	70	0	0

^a^Confidence interval

### Antimicrobial susceptibility

All of the *E. coli* O157:H7 isolates (*n* = 16) were resistant to Amoxicillin while intermediate resistance to Cefoxitine and Nitrofurantoin. All isolates were susceptible to the remaining seven antimicrobials (Table 3).

**Table 3:** Antimicrobial susceptibility of *E. coli* O157:H7 isolates (*n* = 16) obtained from beef cattle at processing plants and retail shops in Ethiopia

Antimicrobial	Susceptible	Intermediate	Resistant
Amoxicillin	0	0	16
Cefoxitin	0	16	0
Chloramphenicol	16	0	0
Ciprofloxacin	16	0	0
Kanamycin	16	0	0
Nalidixic acid	16	0	0
Nitrofurantoin	0	16	0
Streptomycin	16	0	0
Trimethoprim-Sulfamethoxazole	16	0	0
Tetracycline	16	0	0

## Discussion

The aim of the present study was to investigate the occurrence of *E. coli* O157:H7 associated with beef cattle production, processing and distribution, and diarrheal diseases in the public seeking health care services. The prevalence of *E. coli* O157:H7 both in the fecal and carcass swab samples collected both at the processing plants and retail shops was low compared to previous studies conducted in Ethiopia [11–14]. The fact that we observed low prevalence of *E. coli* O157:H7 in the fecal samples (2%) and intestinal mucosa (0.8%) suggests low infection in the beef cattle population studied. This finding can be extrapolated to a larger cattle population of Ethiopia albeit with caution. Although it is difficult to trace back the farms of origin of the cattle slaughtered, it is reasonable to assume that the two processing plants represent wide catchment areas where animals can be brought for slaughter from across the country. Our finding can be confounded by the fact that cattle could be brought both from big commercial feedlots and small scale backyard production systems. Our study suggests the need for large scale on-farm studies to determine the prevalence of *E. coli* O157:H7 under different cattle production systems in Ethiopia. The prevalence of *E. coli* O157:H7 is associated with herd size [18] and it is more common under concentrated animal feeding operations compared to small herds raised typically on pasture [19]. Since the current study was limited only to two beef cattle processing plants, future studies with broader scope representing major beef cattle processing plants in the country are warranted.

The recto-anal junction (RAJ) of cattle is the principal site of colonization for *E. coli* O157: H7 [20] and it was argued that detection of *E. coli* O157: H7 in the intestinal mucosa proximal to RAJ indicates persistent infection by colonization rather than pass through the GIT as detected in the feces [21, 22]. Therefore the low prevalence of *E. coli* O157: H7 in the intestinal mucosal swabs observed in the present study indicates low infection in the beef cattle included in this study. Numerically, we observed higher prevalence of *E. coli* O157: H7 in the fecal samples than in the intestinal mucosal swabs which would suggest rather a passing through of the pathogen than colonization. This was contrary to observation made by Fox et al., [23] in which the prevalence of *E. coli* O157:H7 in the rectal mucosa was twice as much as that observed in the colon content or feces.

The skin of cattle is a significant source for *E. coli* O157: H7 contamination of beef, with the potential for the pathogen transfer onto the carcass during slaughtering and dressing processes [19]. Skin contamination occurs from direct or indirect fecal contamination in beef cattle production and lairage environments; and plays a significant role for downstream carcass contamination. Cross contamination of skins with feces can also occur when a group of cattle is transported or held together in close quarters thus increasing the prevalence of *E. coli* O157: H7 on skins. Level of skin contamination is positively associated with the fecal prevalence of in-coming cattle to the processing plants [19]. Compared to studies reported in other countries, the prevalence (0.91%) of *E. coli* O157: H7 on the skin swabs found in this study is comparatively low. This can be attributed to differences in the factors which can potentially affect skin contamination, including fecal shedding, abattoir management system, farming systems, lairage related conditions, duration of farm/market-to-abattoir transport and hygienic conditions along unloading-to-stunning areas. In this study we note that low prevalence of *E. coli* O157: H7 on skin swabs is expected since its observed prevalence (2% in the fecal samples and 0.8% in the intestinal mucosal swabs) in the cattle population was low. The swabbing site could have an effect on the prevalence of *E. coli* O157: H7 in the skin swab samples. Contrary to other studies [24] which obtained skin swab samples from the shoulder of an animal, we swabbed the ventral surface of the animal over the sternum (brisket) extending over the neck area. This was based on the assumption that as cattle rest in sternal recumbence, this site would be in contact with fecal matter on the ground thus maximizing skin contamination. Also, since our swabbing site included the bleeding site this would facilitate entry of the pathogen to carcass surface during slaughtering process. Even though our sampling method (using cotton tipped swabs instead of sponges) and selection of swabbing site (ventral part of the animal) could have contributed to the low apparent prevalence of *E. coli* O157: H7 in the skins samples, this effect can be considered minimal compared to the impact of low overall prevalence of *E. coli* O157: H7 observed in the cattle population studied.

We observed low level of carcass contamination by *E. coli* O157: H7 at the processing plants (0.54%) or retail shops (0.43%). This can be attributed generally to the low prevalence of *E. coli* O157 observed in the cattle population (fecal, intestinal mucosal swabs and skin swab samples) as well as on the skins. Even though the prevalence of *E. coli* O157: H7 was low, numerically we observed a gradual decline of the prevalence from the fecal (2%), skin (0.9%) and carcass samples (0.5%) at the processing plants demonstrating that current sanitary dressing procedures are effective against *E. coli* O157: H7. It can also indicate good sanitary procedures observed at the processing plants and the retail shops. Our results are lower compared to previous studies conducted in Ethiopia. For instance *E. coli* O157: H7 was reported in 8% of beef samples collected at abattoirs and retail shops [12], in 2.7% of beef carcass swab samples collected from a slaughter house [25], and in 2.1% of beef carcass and cutting board swab samples collected from retailer shops [26]. Carcass contamination by feces can occur directly from the intestinal content during evisceration, or indirectly from skins during dressing operation or from the abattoir environment such as from contact with conveyer belts. Lack of detection of *E. coli* O157:H7 in the environmental samples could be attributed to the low overall prevalence of this pathogen in the present study and the small number of environmental samples (*n* = 62).

*E. coli* O157: H7 was not detected in the stool samples from people with diarrhea seeking health services. Studies reporting *E. coli* O157: H7 in humans are limited in Ethiopia despite the common occurrence of diarrhea problems especially in children. One study [11] conducted in children (*n* = 422) under five years of age with acute diarrhea reported 14% prevalence of *E. coli* O157: H7. Since our sample size (70 cases) was not sufficient, compared to the aforementioned study (422 cases), to draw conclusive evidence we recommend a more population based study. The human clinical *E. coli* O157: H7 isolates (*n* = 59) in the above study [11] exhibited resistance, at varying degrees, to all of the antimicrobials tested with the highest resistance (90%) to ampicillin. Among the *E. coli* O157: H7 isolates from beef cattle, skins and carcass samples, even though antimicrobial resistance was rare, all the isolates were resistant to ampicillin. Similarly, Taye et al. [25] reported 100% resistance to Ampicillin and Amoxicillin. The high prevalence of resistance to the beta-lactam class of antimicrobials both in the clinical and animal origin *E. coli* O157: H7 isolates requires further investigation. Even though it is impossible to draw epidemiologic association between the observed high resistance to the beta-lactam classes of antimicrobials and the frequency (quantity) of use of these antimicrobials in this study, we speculate that beta-lactams could be the most commonly used antimicrobials both in humans and cattle in Ethiopia. This hypothesis needs to be further elucidated since determining the amount of antibiotic use both in humans and animals is critical to assess the contribution of antibiotic use to the level of AMR observed in a given country.

## Conclusion

*E. coli* O157: H7 was identified at low prevalence from the feces, intestinal mucosal swab and skin swab samples collected from cattle slaughtered at processing plants. Isolation of *E. coli* O157: H7 from the fecal samples and intestinal mucosal swabs indicates carriage of the pathogen by the animal. Although the prevalence of *E. coli* O157: H7 is low, its public health impact should not be underestimated given its low infective dose. The present study showed that the prevalence of *E. coli* O157: H7 in beef cattle production setting is low; current sanitary processing procedures at the processing plants; and good carcass handling at the retail shops are effective against *E. coli* O157: H7. Further large scale epidemiological studies in the beef cattle production and processing continuum are recommended to further substantiate our present findings.
